# Supplementary material for: Modeling glioblastoma heterogeneity as a dynamic network of cell states
Source: Mol Syst Biol. 2021 Sep 16;17(9):e10105. doi: 10.15252/msb.202010105 (PMC8444284; doi:10.15252/msb.202010105)
Supplement: Supplementary file 6 — Source Data for Figure 5 [file MSB-17-e10105-s004.zip › Figure5A_sourcedata/GSEA_3071/hallmarks_stateI.GseaPreranked.1623742531165/gsea_report_for_na_pos_1623742531165.html]

Report for na\_pos 1623742531165 [GSEA]

| GS  follow link to MSigDB | GS DETAILS | SIZE | ES | NES | NOM p-val | FDR q-val | FWER p-val | RANK AT MAX | LEADING EDGE || 1 | HALLMARK\_EPITHELIAL\_MESENCHYMAL\_TRANSITION | Details ... | 116 | 0.58 | 3.54 | 0.000 | 0.000 | 0.000 | 457 | tags=54%, list=13%, signal=60% |
| 2 | HALLMARK\_ANGIOGENESIS | Details ... | 19 | 0.77 | 3.02 | 0.000 | 0.000 | 0.000 | 255 | tags=63%, list=7%, signal=68% |
| 3 | HALLMARK\_INTERFERON\_ALPHA\_RESPONSE | Details ... | 36 | 0.61 | 2.88 | 0.000 | 0.000 | 0.000 | 1030 | tags=83%, list=29%, signal=117% |
| 4 | HALLMARK\_INTERFERON\_GAMMA\_RESPONSE | Details ... | 61 | 0.51 | 2.68 | 0.000 | 0.000 | 0.000 | 1030 | tags=70%, list=29%, signal=98% |
| 5 | HALLMARK\_IL2\_STAT5\_SIGNALING | Details ... | 66 | 0.48 | 2.61 | 0.000 | 0.000 | 0.000 | 410 | tags=36%, list=12%, signal=40% |
| 6 | HALLMARK\_COAGULATION | Details ... | 40 | 0.50 | 2.40 | 0.000 | 0.000 | 0.001 | 352 | tags=43%, list=10%, signal=47% |
| 7 | HALLMARK\_ADIPOGENESIS | Details ... | 53 | 0.46 | 2.37 | 0.000 | 0.000 | 0.001 | 605 | tags=45%, list=17%, signal=54% |
| 8 | HALLMARK\_MYOGENESIS | Details ... | 61 | 0.42 | 2.22 | 0.000 | 0.001 | 0.002 | 371 | tags=38%, list=11%, signal=41% |
| 9 | HALLMARK\_APOPTOSIS | Details ... | 66 | 0.37 | 2.01 | 0.000 | 0.004 | 0.021 | 623 | tags=41%, list=18%, signal=49% |
| 10 | HALLMARK\_P53\_PATHWAY | Details ... | 67 | 0.36 | 1.98 | 0.000 | 0.004 | 0.024 | 474 | tags=31%, list=13%, signal=36% |
| 11 | HALLMARK\_COMPLEMENT | Details ... | 60 | 0.37 | 1.94 | 0.000 | 0.005 | 0.032 | 585 | tags=40%, list=17%, signal=47% |
| 12 | HALLMARK\_UV\_RESPONSE\_DN | Details ... | 61 | 0.36 | 1.92 | 0.006 | 0.006 | 0.036 | 446 | tags=30%, list=13%, signal=33% |
| 13 | HALLMARK\_BILE\_ACID\_METABOLISM | Details ... | 16 | 0.51 | 1.89 | 0.005 | 0.008 | 0.054 | 868 | tags=69%, list=25%, signal=91% |
| 14 | HALLMARK\_ALLOGRAFT\_REJECTION | Details ... | 39 | 0.39 | 1.85 | 0.003 | 0.009 | 0.070 | 407 | tags=33%, list=12%, signal=37% |
| 15 | HALLMARK\_GLYCOLYSIS | Details ... | 68 | 0.33 | 1.81 | 0.006 | 0.012 | 0.091 | 573 | tags=34%, list=16%, signal=40% |
| 16 | HALLMARK\_REACTIVE\_OXYGEN\_SPECIES\_PATHWAY | Details ... | 23 | 0.43 | 1.79 | 0.018 | 0.014 | 0.115 | 671 | tags=57%, list=19%, signal=69% |
| 17 | HALLMARK\_APICAL\_JUNCTION | Details ... | 67 | 0.32 | 1.73 | 0.006 | 0.019 | 0.167 | 660 | tags=39%, list=19%, signal=47% |
| 18 | HALLMARK\_UNFOLDED\_PROTEIN\_RESPONSE | Details ... | 51 | 0.33 | 1.70 | 0.000 | 0.024 | 0.219 | 1115 | tags=55%, list=32%, signal=79% |
| 19 | HALLMARK\_TGF\_BETA\_SIGNALING | Details ... | 22 | 0.41 | 1.68 | 0.033 | 0.026 | 0.239 | 682 | tags=50%, list=19%, signal=62% |
| 20 | HALLMARK\_INFLAMMATORY\_RESPONSE | Details ... | 52 | 0.31 | 1.60 | 0.028 | 0.039 | 0.347 | 478 | tags=31%, list=14%, signal=35% |
| 21 | HALLMARK\_HYPOXIA |  | 72 | 0.28 | 1.56 | 0.022 | 0.049 | 0.427 | 888 | tags=46%, list=25%, signal=60% |
| 22 | HALLMARK\_MTORC1\_SIGNALING |  | 92 | 0.25 | 1.45 | 0.023 | 0.092 | 0.675 | 875 | tags=40%, list=25%, signal=52% |
| 23 | HALLMARK\_PROTEIN\_SECRETION |  | 33 | 0.30 | 1.37 | 0.121 | 0.128 | 0.803 | 1393 | tags=73%, list=40%, signal=119% |
| 24 | HALLMARK\_IL6\_JAK\_STAT3\_SIGNALING |  | 18 | 0.36 | 1.36 | 0.153 | 0.132 | 0.832 | 1215 | tags=67%, list=35%, signal=101% |
| 25 | HALLMARK\_HEME\_METABOLISM |  | 39 | 0.28 | 1.32 | 0.138 | 0.154 | 0.876 | 592 | tags=36%, list=17%, signal=43% |
| 26 | HALLMARK\_TNFA\_SIGNALING\_VIA\_NFKB |  | 67 | 0.22 | 1.19 | 0.207 | 0.259 | 0.975 | 872 | tags=43%, list=25%, signal=56% |
| 27 | HALLMARK\_XENOBIOTIC\_METABOLISM |  | 47 | 0.23 | 1.15 | 0.269 | 0.299 | 0.987 | 623 | tags=36%, list=18%, signal=43% |
| 28 | HALLMARK\_ESTROGEN\_RESPONSE\_EARLY |  | 70 | 0.20 | 1.09 | 0.350 | 0.363 | 0.994 | 898 | tags=39%, list=26%, signal=51% |
| 29 | HALLMARK\_ANDROGEN\_RESPONSE |  | 36 | 0.22 | 1.04 | 0.413 | 0.434 | 1.000 | 438 | tags=25%, list=12%, signal=28% |
| 30 | HALLMARK\_CHOLESTEROL\_HOMEOSTASIS |  | 22 | 0.24 | 0.97 | 0.494 | 0.522 | 1.000 | 392 | tags=27%, list=11%, signal=31% |
| 31 | HALLMARK\_PI3K\_AKT\_MTOR\_SIGNALING |  | 36 | 0.19 | 0.89 | 0.619 | 0.635 | 1.000 | 412 | tags=22%, list=12%, signal=25% |
Table: Gene sets enriched in phenotype **na **[plain text format]****
